# Prenatal stress induced depressive‐like behavior and region dependently high CRP level in offspring rats

**DOI:** 10.1002/brb3.2046

**Published:** 2021-02-18

**Authors:** Shaoning Li, Huifang Zhang, Xueyun Gao, Huimei Huang, Wei He, Huiping Zhang, Hongli Sun

**Affiliations:** ^1^ Department of Emergency Xi’an Children’s Hospital The Affiliated Children’s Hospital of Xi’an Jiaotong University) Xi’an, Shaanxi P.R. China; ^2^ School of Public Health Xi’an Jiaotong University Xi’an, Shaanxi P.R. China; ^3^ Department of Nephrology Xi’an Children’s Hospital The Affiliated Children’s Hospital of Xi’an Jiaotong University) Xi’an, Shaanxi P.R. China; ^4^ Shaanxi Institute for Pediatric Diseases Xi'an Key Laboratory of Children's Health and Diseases Xi’an Children’s Hospital The Affiliated Children’s Hospital of Xi’an Jiaotong University) Xi’an, Shaanxi P.R. China

**Keywords:** C‐reactive protein, depressive‐like behavior, prenatal stress

## Abstract

**Introduction:**

To explore the changes in C‐reactive protein (CRP) level in different regions of one old offspring rats exposed to prenatal stress (PS).

**Methods:**

The rat model was constructed with prenatal restraint stress on pregnant dams on days 14–20 of gestation. Offspring rats were randomly divided into PS susceptibility (PS‐S) group and control (CON) group. Behavioral experiments including sucrose preference test (SPT), open‐field test (OFT), and forced swimming test (FST) were used to measure depressive‐like behaviors. Immunohistochemistry, qRT‐PCR, and Western blotting were applied to detect the changes in CRP level.

**Results:**

The results showed that PS could cause depressive‐like behaviors in all SPT, OFT, and FST. Concomitantly, CRP mRNA and protein expression significantly increased in hippocampus, prefrontal cortex, and hypothalamus in the PS‐S group when compared that in the CON group, while no significantly changes in liver, heart, olfactory bulb, striatum, and cerebellum in the PS‐S group when compared that in the CON group.

**Conclusion:**

Increasing of CRP expression in hippocampus, prefrontal cortex, and hypothalamus may play a critical role in the mechanism under depressive‐like behavior in offspring rats exposed to PS.

## Graphical abstract

Prenatal stress (PS) induced depressive‐like behavior in susceptible offspring rats, and an increasing of CRP expression in hippocampus, prefrontal cortex, and hypothalamus. Thus, higher CRP expression in hippocampus, prefrontal cortex, and hypothalamus may play a critical role in the mechanism under depressive‐like behavior in offspring rats exposed to PS.

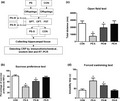

## 1 INTRODUCTION

Prenatal stress that can cause depression or depressive‐like behaviors had been verified by a large number of studies including clinical and laboratory researches. In humans, it was reported that prenatal stress could cause neurodevelopment, cognitive development, negative affectivity, and difficult temperament, which are all high risks for subsequent depression in life (Lautarescu et al., 2020; Van den Bergh et al., 2017), even more directly cause depression symptoms in adolescent (Murphy et al., 2017). These depressive symptoms had also been imitated in animal experiments of our and other research teams, which showed depressive‐like behaviors including extended immobile time in forced swimming test (FST), reduced total number of grids explored, number of times, the center grid was crossed, number of times rat stood vertically erect, and time spent in the central grid in open‐field test (OFT), decreased percentage preference for sucrose in sucrose preference test (SPT) (Lu et al., 2020; Wang et al., 2020). Under the mechanism of these clinical phenomena and experimental results, a recent review suggested that neuroinflammation is a key factor that interacts with neurobiological correlates of major depressive disorder and neuroinflammation may be a key therapeutic target for future therapeutic strategies in major depressive disorder (Troubat et al., 2020).

C‐reactive protein (CRP) is a general marker of peripheral inflammation and has been shown to be a good marker of neuroinflammation (Fond et al., 2020). CRP is a pentameric protein synthesized by the liver, whose level rises in response to inflammation (Nehring et al., 2020). Numerous clinical studies have shown that a significantly higher circulating CRP level was involved in patients with depression (Iob et al., 2020; Osimo et al., 2019; Wang et al., 2018) and has a lower antidepressants efficacy (Li et al., 2019; Zhang, Yue, et al., 2019). Some other works have showed that heart diseases, cancer patients with depressive symptoms also had a higher CRP level (McFarland et al., 2019; Ravona‐Springer et al., 2020). However, there were also conflicting findings showed that CRP was not associated with depressive symptoms nor response to antidepressant treatment (Miller et al., 2019; Zhang et al., 2018). These conflicting results require validation with a larger clinical sample and more profound investigation at the animal level.

Hippocampus, prefrontal cortex, hypothalamus, olfactory bulb, striatum, and cerebellum were reported as susceptible brain areas under depression, and these regions were not only involved in the mechanism of depression but the antidepressant treatment (Huang et al., 2020; Xu et al., 2020; Yuan et al., 2020; Zhang et al., 2020). To some extent, the complex pathogenesis of depression may be a result of a disturbance of the network connection function of brain region. Thus, in present study, we preliminarily measured the effect of PS on the change of CRP in liver, heart, hippocampus, prefrontal cortex, hypothalamus, olfactory bulb, striatum, and cerebellum of offspring rats trying to explain the depressive‐like behavior in offspring rats caused by PS.

## 2 MATERIALS AND METHODS

### 2.1 Animal subjects

**FIGURE 1:**
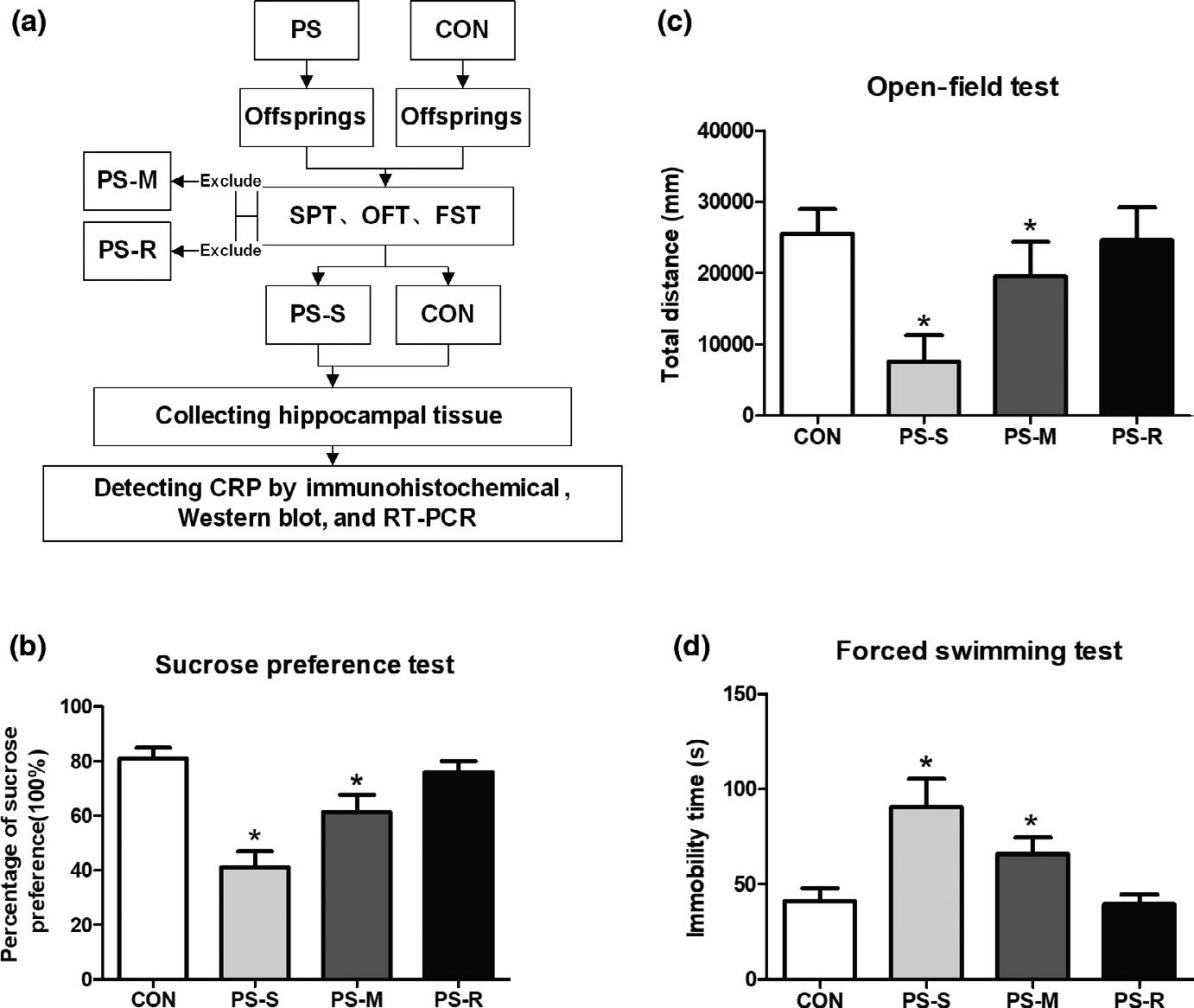
Schematic workflow of the experimental design (a) and behavioral assessment of the PS rat model. (b) Percentage of sucrose preference in SPT (*n* = 15 per group). (c) Total distance in OFT (*n* = 8 per group). (d) Immobility time in FST (*n* = 8 per group). Values represent means ± *SD*, *p < .05 versus CON. PS: prenatal stress; CON: control; PS‐S: PS susceptibility; PS‐M: PS midterm; PS‐R: PS resistance; SPT: sucrose preference test; OFT: open‐field test; FST: forced swimming test; CRP: C‐reactive protein

Sprague‐Dawley rats were used in present study. During the experiment, all rats were maintained under a 12‐hr dark/12‐hr light cycle at 22℃ and allowed to drink and eat ad libitum excepting during periods of stress. Six adult female rats and two adult male rats mated by 3:1 at 20:00 ~ 22:00 pm. In the next morning, positive sperms in vagina of female rats were detected as gestational day (GD) 0. Pregnant dams were raised in a single cage. All experimental procedures were approved by the experimental animal care and use committee of Xi'an Jiaotong University. The flow chart of the experiment was shown in Figure 1a.

### 2.2 PS procedure

Prenatal restraint stress procedure was implemented according to the previous article (Koehl et al., 2015). Briefly, pregnant dams were randomly divided into prenatal stress (PS) and control (CON) groups. PS dams were placed into a plastic bottle with an adjustable cap to keep the dam's head still (3 times/day, 45 min/time). Dams from the CON group were placed to the same situation but without stress procedure. After both groups, dams gave birth and the pups were weaned. All offspring rats were subjected to behavioral tests to distinguish PS susceptibility (PS‐S), PS midterm (PS‐M), and PS resistance (PS‐R) groups, and CON offspring rats were collected from the CON dams. A total of 60 (no <6 in each group) offspring rats were used in present experiment.

### 2.3 Sucrose preference test

Sucrose preference test was implemented according to a previous report (Henningsen et al., 2012). Briefly, all offspring rats were subjected to SPT when 1 month old. At firstly, rats were habituated to a bottle with 1% sucrose solution for 24 hr. Then, rats were deprived of food and water for 24 hr. During the test, a bottle of normal tap water and a bottle of 1% sucrose solution were provided to the rats simultaneously. After 1 hr, the remaining normal tap water and sucrose solution were measured and the percentage of sucrose preference was calculated. The algorithm was as follows: the percentage of sucrose preference = (sucrose consumption/(sucrose consumption + normal tap water consumption) × 100%). When compared to the average of the percentage of sucrose preference in the CON group, the reduction was more than 30% was assigned to the PS‐S group, less than 10% that was assigned to the PS‐R group, between 10%~30% that was assigned to the PS‐M group.

### 2.4 Open‐field test

After SPT, rats were subjected to OFT (Taimeng software Co. Ltd, Item No: OFT‐100, Chengdu, China). Briefly, an open‐field apparatus with 74 cm in width, 62 cm in length, and 51 cm in height. The bottom of the box was divided into 25 equal squares, and rats were placed down within the center nine grids. The OFT was located at a sound‐attenuating chamber and illuminated with 20 lux light. The total distance of rat's activity was recorded by a video camera within 5 min. Feces and urine on the bottom were cleaned by alcohol to avoid the residual smell from every rat.

### 2.5 Forced swimming test

After OFT, rats were subjected to FST (Youcheng jiaye biotechnology Co. Item No: LTDLE803/804, Beijing, China). Briefly, a vertical glass cylinder with 50 cm in height and 20 cm in diameter, and the cylinder was filled with water with 30 cm in height and its temperature was maintained at 30℃. All rats were habituated to the cylinder for 15 min before experiment. After 24 hr, rats were placed into the cylinder again and the immobile time was recorded within 5 min by video analysis. The immobile time was defined as the total time of no struggling, or only minimal movements during swimming. After the termination of all behavioral tests, rats were anesthetized with sodium pentobarbital (60 mg/kg, i.p.), and the liver, heart, hippocampus, prefrontal cortex, hypothalamus, olfactory bulb, striatum, and cerebellum tissues were sectioned and homogenized on ice.

### 2.6 Immunohistochemistry

Immunohistochemical staining for CRP was carried out in 4‐μm tissue sections prepared from formalin‐fixed, paraffin‐embedded tissue blocks. All specimens were subjected in turn to rehydration, antigen retrieval, and endogenous peroxidase activity block in 3% H_2_O_2_; then, a Pap pen was used to create a hydrophobic barrier around each tissue sections. After blocked with 5% (w/v) goat serum albumin for 30 min at room temperature, mouse monoclonal anti‐CRP antibody (1:200, 66250‐1‐Ig, ProteinTech Group, Inc) was used at recommended concentration of the instruction for IHC staining at 4°C overnight followed by secondary antibody (Scicrest Biotech, 1:200) for 30 min at 37°C. Diaminobenzidine (DAB, ZLI‐9018, Zhong Shan Jin Qiao) was employed for 5 min as the chromogen. Hematoxylin was employed for counterstaining. The numbers of positive cells or relative intensity in five sections per rat from six rats were quantitated and averaged by Image‐Pro Plus 6.0 software.

### 2.7 Quantitative reverse‐transcription polymerase chain reaction (QRT‐PCR)

Total RNA was extracted using TRIzol (Aidlab Biotechnologies), and a NanoDrop ND‐100 spectrophotometer (NanoDrop Technologies) was used to quantify RNA concentration with absorbance at 260 nm. Isolated RNA was transcribed into cDNA using the Omniscript® Reverse Transcriptase kit (Vazyme Biotech), and an ABI 7900HT or QuantStudio 6 System (Applied Biosystems) was used to perform qRT‐PCR. The primer sequences used for qRT‐PCR were as follows: CRP, 5′‐GTCTCTATGCCCACGCTGAT‐3′ (F), 5′‐CCGTCAAGCCAAAGCTCTAC‐3′ (R); β‐actin, 5′‐CACGATGGAGGGGCCGGACTCATC‐3′ (F), 5′‐TAAAGACCTCTATGCCAACACAGT‐3′ (R). β‐Actin was used as a normalization control, and the 2^−ΔΔCt^ method was used for quantification of relative expression. All reactions were performed in triplicate.

### 2.8 Western blotting

Hippocampus tissue was sectioned and homogenized on ice in RIPA lysis buffer with phosphatase enzyme inhibitor cocktail (Beyotime Ins. Biotec). Lysates were centrifuged at 12,000 *g* for 5 min, and protein concentration was measured by a BCA kit (Beyotime Ins. Biotec). Lysate samples (20 mg) were separated using 5% and 10% SDS‐PAGE, transferred to a porous polyvinylidene fluoride (PVDF) membrane (Millipore). The primary antibodies used were as follows: mouse monoclonal anti‐CRP antibody (1:2000; 66250–1‐ap, Wuhan Sanying Biotechnology Co. LTD) and mouse monoclonal anti‐β‐actin (1:500; BM0627, Boster Biological Technology co.ltd) were used as an internal loading control. SuperSignal® West Dura Extended Duration Substrate (Pierce) and X‐ray Film (Kodak) were used for detection, and intensities were quantified using Bandscan 5.0 software (Funglyn Biotech).

### 2.9 Statistical analysis

Data were presented as means ± standard deviation (*SD*). Kolmogorov–Smirnov was used for normality test. Student's *t* test or one‐way ANOVA was used for comparison between groups. Mann–Whitney and Kruskal–Wallis were used when the data do not conform to a normal distribution. *p* < .05 was set as a significant level. Statistical analysis was accomplished by Prism version 5.0 software (GraphPad Software Inc., San Diego, CA).

## 3 RESULTS

### 3.1 Assessment of depressive‐like behavior in offspring rats under PS rat model

SPT, OFT, and FST were employed to evaluate depressive‐like behavior in offspring rats exposed to PS. At firstly, SPT was used to distinguish the responses of offspring rats to PS. The results showed that the percentage of sucrose preference significantly decreased in the PS‐S or PS‐M group when compared that in the CON group (all *p* < .05, Student's *t* test), while no significantly changes in the PS‐R group (*p* > .05, Student's *t* test) (Figure 1b). For the results of OFT, data showed that total distance of rat's activity from the PS‐S or PS‐M group significantly decreased when compared that in the CON group (all *p* < .05, Student's *t* test), while no significantly changes in the PS‐R group (*p* > .05, Student's *t* test) (Figure 1c). For the results of FST, data showed that immobility time or rats from the PS‐S or PS‐M group significantly increased when compared that in the CON group (all *p* < .05, Student's *t* test), while no significantly changes in the PS‐R group (*p* > .05, Student's *t* test) (Figure 1d). These data manifested that prenatal restraint stress is a valid model to induce depressive‐like behavior in rats, and SPT is a viable tool for distinguish the responses of offspring rats to PS.

### 3.2 Immunohistochemistry showed the effect of PS on CRP protein expression in different tissues of offspring rats

**FIGURE 2:**
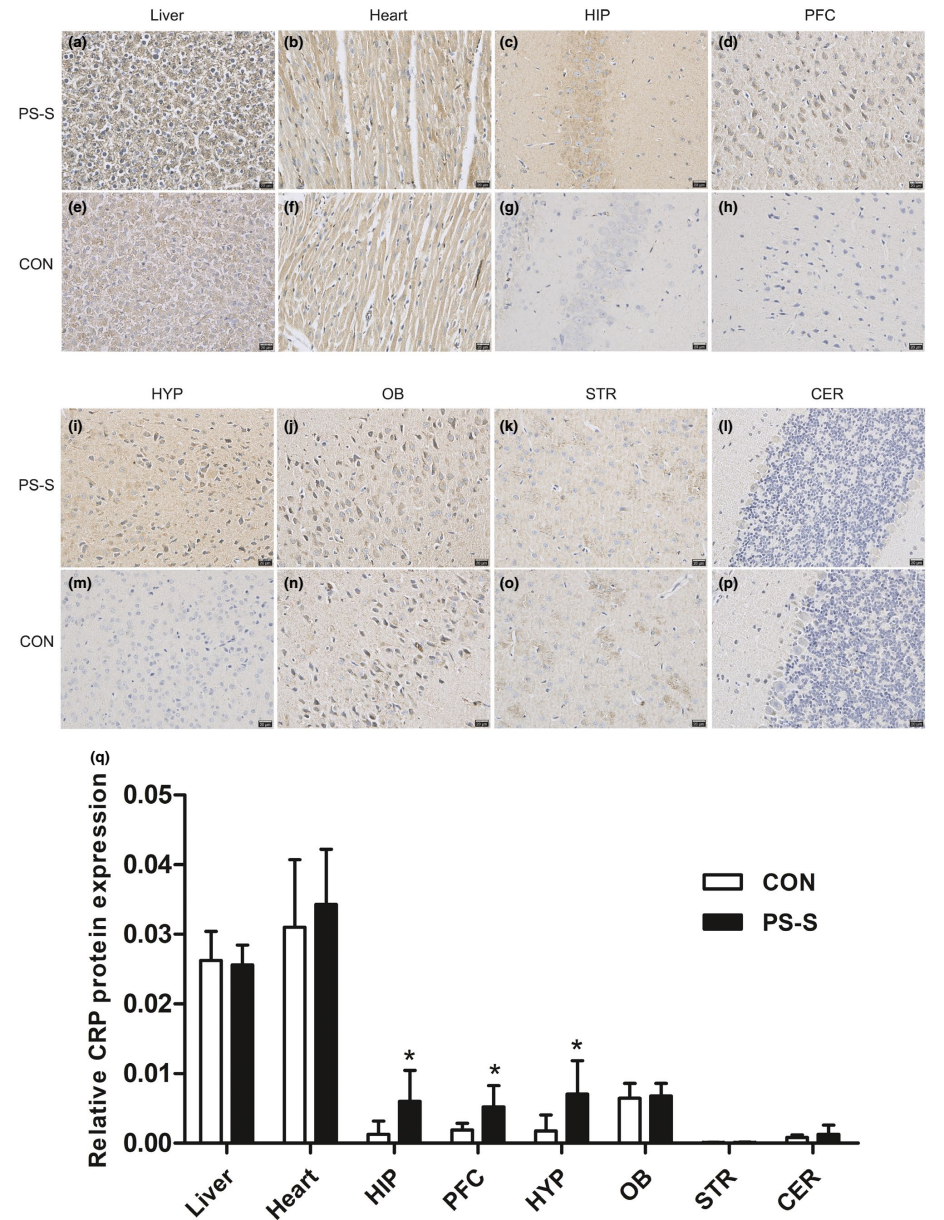
Representative immunohistochemistry showed CRP protein expression in different regions of rats from the two groups. Immunohistochemistry images of CRP expression in liver in the PS‐S (a) and CON (e) groups, heart in the PS‐S (b) and CON (f), HIP in the PS‐S (c) and CON (g) group, PFC in the PS‐S (d) and CON (h) groups, HYP in the PS‐S (i) and CON (m) groups, OB in the PS‐S (j) and CON (n) groups, STR in the PS‐S (k) and CON (o) groups, CER in the PS‐S (l) and CON (p) groups. Blue represents the cell nucleus and brownish‐yellow or tan represents the CRP protein (Original magnification 400×). The quantitative statistical map of immunohistochemistry (q) (*n* = 6 per group). CON: control; PS‐S: PS susceptibility; CRP: C‐reactive protein. HIP: hippocampus; PFC: prefrontal cortex; HYP: hypothalamus; OB: olfactory bulb; STR: striatum; CER: cerebellum

To show a more significant effect of PS on behavioral and molecular mechanisms of the offspring rats, we next used the PS‐S rats with more dramatically behavioral changes for the detection of CRP levels changes. As showed in Figure 2, CRP protein expression significantly increased in hippocampus, prefrontal cortex, and hypothalamus in the PS‐S group when compared that in the CON group (all *p* < .05, Student's *t* test), while no significantly changes in liver, heart, olfactory bulb, striatum, and cerebellum in the PS‐S group when compared that in the CON group (all *p* > .05, Student's *t* test). These data manifested that increasing of CRP expression in hippocampus, prefrontal cortex, and hypothalamus may play a critical role in the mechanism under depressive‐like behavior in offspring rats exposed to PS.

### 3.3 Changes in CRP mRNA and protein expression levels caused by PS

**FIGURE 3:**
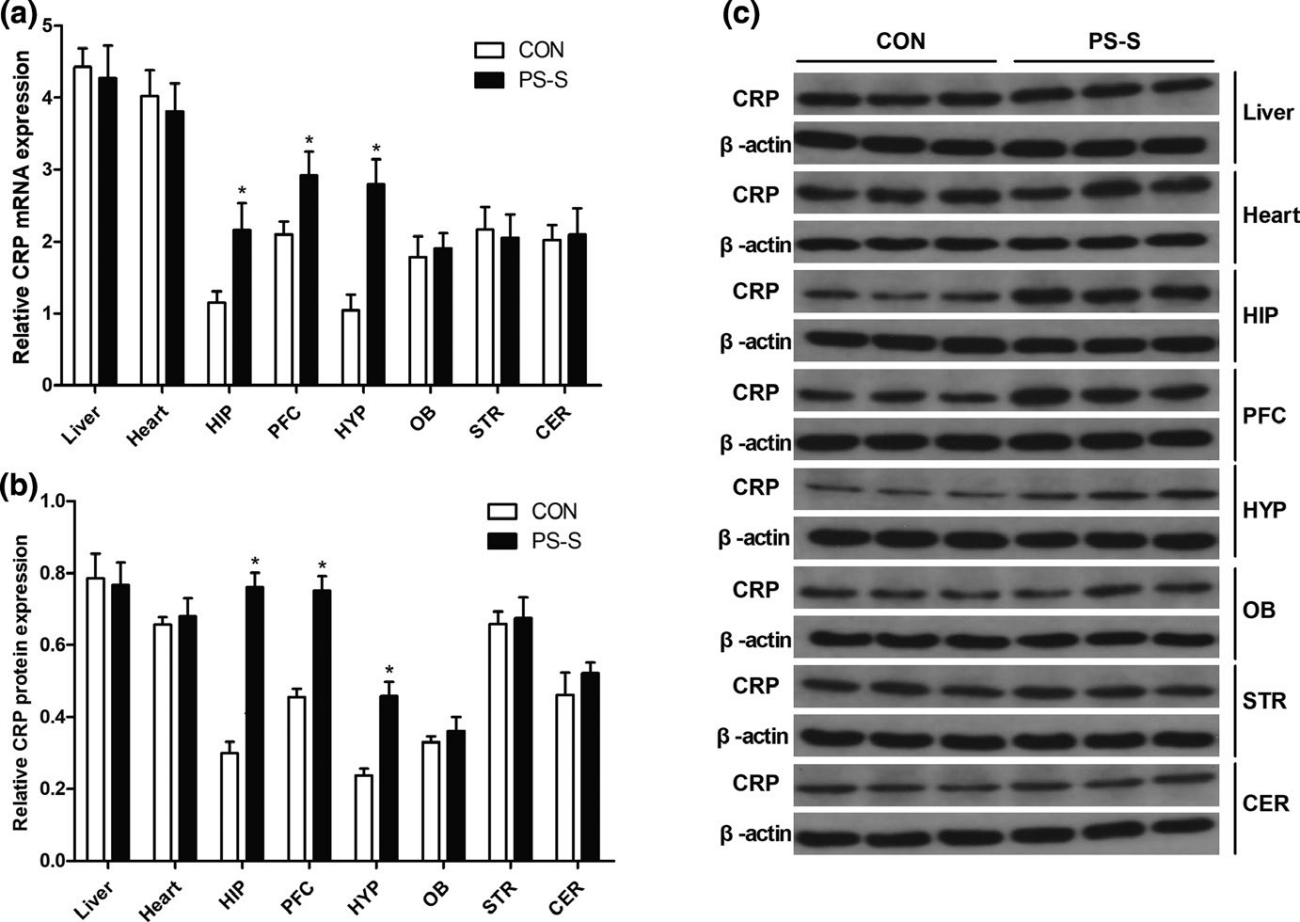
Levels of CRP mRNA and protein expression by RT‐PCR and Western blotting. (a) Levels of CRP mRNA in different tissues in both PS‐S and CON groups (*n* = 6 per group). (b) Levels of CRP protein expression in different tissues in both PS‐S and CON groups (*n* = 6 per group). (c) Bands of CRP protein expression in different tissues in both PS‐S and CON groups (*n* = 6 per group). Mean ± *SD*, *p < .05

To confirm the changes in CRP expression of PS‐S rats, we next used RT‐PCR and Western blotting to detect the changes in CRP mRNA and protein expression levels in offspring rats caused by PS. Paralleled to those of immunohistochemistry, CRP mRNA levels in hippocampus, prefrontal cortex, and hypothalamus in the PS‐S group were significantly higher than that in the CON group (all *p* < .05, Student's *t* test), while no significantly changes in liver, heart, olfactory bulb, striatum, and cerebellum in the PS‐S group when compared that in the CON group (all *p* > .05, Student's *t* test) (Figure 3a). Correspondingly, CRP protein expression in hippocampus, prefrontal cortex, and hypothalamus in the PS‐S group was also significantly higher than that in the CON group (all *p* < .05, Student's *t* test), while no significantly changes in liver, heart, olfactory bulb, striatum, and cerebellum in the PS‐S group when compared that in the CON group (all *p* > .05, Student's *t* test) (Figure 3b,c). These data confirmed the results of immunohistochemistry that increasing of CRP expression in hippocampus, prefrontal cortex, and hypothalamus may play a critical role in the mechanism under depressive‐like behavior in offspring rats exposed to PS.

## 4 DISCUSSION

In present work, we found that PS could cause depressive‐like behavior in susceptible offspring rats. Correspondingly, PS also caused a significantly increasing CRP level in hippocampus, prefrontal cortex, and hypothalamus in susceptible offspring rats when compared its matched controls.

Susceptibility and resistance are two different types of response to stress and also are a common clinical phenomenon. Many literatures have provided the molecular basis of this mechanism (Bagot et al., 2017; Henningsen et al., 2012; Tang et al., 2019; Zhang, He, et al., 2019). For example, Xie Peng and his colleagues used the mass spectrometry and the isobaric tags for relative and absolute quantitation (iTRAQ) labeling technique found that there were 367 hippocampal protein candidates might be associated with susceptibility to stress‐induced depression or anxiety and stress resilience (Tang et al., 2019). Transcriptional profiles have been changed dramatically between susceptibility and resilience in the prefrontal cortex of mice exposed to chronic social defeat stress, and these changes could be reversed by antidepressants including imipramine and ketamine (Bagot et al., 2017). In our previous work, we monitored changes in hippocampus metabolites during the development of depressive‐like behaviors in rats exposed to PS via UHPLC‐Q‐TOF/MS approach and we found that a total of 38 differential metabolites were detected in the susceptibility rats exposed to PS compared with that in controls (Zhang, He, et al., 2019). These literatures provide a good theoretical basis for us to search for molecular targets in the future.

SPT as a well‐recognized evaluation tool of depression and it can effectively detect the presence or absence of anhedonia, which is the core symptom of depression (Overstreet, 2012; Yan et al., 2010). We have previously reported that offspring rats exposed to PS show a graded sucrose‐intake, while no concurrent reduction in water intake (Sun et al., 2017; Wang et al., 2020; Zhang, He, et al., 2019). These changes were also confirmed by many other literatures using chronic mild stress rat model (Bisgaard et al., 2007; Henningsen et al., 2012). The data of sucrose consumption follow a right‐skewed normal distribution, and thus, the definition as stress‐susceptible and stress‐resilient rats, respectively, is based on an operational cutoff chosen by the experimenter. Thus, SPT could be a valid tool for distinction into stress‐susceptible and stress‐resilient rats.

Higher CRP levels in rats showed depressive‐like behavior were found consistently with most previous reports (Felger et al., 2020; Tab atabaeizadeh et al., 2018). Inflammation can also lead to the activation of the tryptophan (TRP)‐degrading enzyme indoleamine 2,3 dioxygenase (IDO) (Maes et al., 2011), ultimately increasing the formation of kynurenine (KYN) metabolites, including kynurenic acid (KA), a putatively neuroprotective antagonist of N‐methyl‐d‐aspartate (NMDA) receptors that also decreases glutamate levels via inhibition of α7 nicotinic receptors 3‐hydroxykynurenine (3HK), a free radical generator, and quinolinic acid (QA), an NMDA receptor agonist that also exerts neurotoxic effects via lipid peroxidation, and disruption of the blood–brain barrier (Dantzer et al., 2011; Savitz et al., 2015; Schwarcz et al., 1983, 2012). Inflammation is also known to influence glutamate neurotransmission by binding to its NMDA receptors, leading to chaotic, noisy, incoherent signaling activity in the short‐term, and synaptic toxicity by suppressing intracellular survival mechanisms in the long‐term (Hardingham & Bading, 2010; Haroon et al., 2018). Our previous work has indicated that PS could increase glutamate level in hippocampus (Zhang, He, et al., 2019), decrease NMDA subunit NR1 and NR2 (Sun et al., 2013) and decrease postsynaptic density (PSD)‐95 which was normalized by antidepressant treatment (Wang et al., 2020). Our previous work also manifested that the key receptor of endocannabinoid system CB1 and the key receptor of glucocorticoid system GR were also changed in offspring rats exposed by PS (Sun et al., 2017; Wang et al., 2020; Zhang, He, et al., 2019). Thus, our present work extended the hypothesis that neuroinflammation, neurotransmitter, and endocrine system were co‐involvement in the pathogenesis of depression.

A large‐scale network‐based analyses of resting‐state functional magnetic resonance imaging (rfMRI) data showed that increased plasma CRP was associated with reduced functional connectivity in a widely distributed network including ventral striatum, parahippocampal gyrus/amygdala, orbitofrontal and insular cortices, and posterior cingulate cortex in patients with depression (Yin et al., 2019). Hippocampus is known as one of the most sensitive‐stressed regions related to depression. Neuroinflammation of the hippocampus was involved in the depression and antidepressant treatment (Yue et al., 2018). Animal studies indicated that chronic stress can cause an increase in IL‐1β level in the hippocampus and prefrontal cortex (Liu et al., 2013; Pan et al., 2014). Zili You and his colleagues have found that maternal infection, maternal sleep disturbance during pregnancy, and maternal separation upregulated pro‐inflammatory markers and downregulated anti‐inflammatory markers in the hippocampus of offsprings, and they activated microglia and promoted pro‐inflammatory transitions in microglia (Han et al., 2019, 2020; Zhao et al., 2019). The activation of peroxisome proliferator‐activated receptor gamma, which is a ligand‐dependent transcription factor in the nuclear hormone receptor family that has been known to mediate immune inflammatory responses, is like minocycline that could exert neuroprotective effects by regulating neuroinflammatory response (Han et al., 2019, 2020; Zhao et al., 2019). These results showed that PPARγ may serve as a potential therapeutic approach for prenatal immune activation‐induced neuropsychiatric disorders. Here, we first reported that CRP levels in hippocampus, prefrontal cortex, and hypothalamus were associated with depressive‐like behavior in offspring rats exposed to PS. According to the above reports, it can be seen that inflammation state in the hippocampus, prefrontal cortex, and hypothalamus may be play an essential role in the pathogenesis of depressive‐like behavior in offspring rats exposed to PS. Our present work supports the hypothesis that inflammation as a distinct contributing factor to network dysfunction and symptom severity in depression.

A large number of literature reports indicated that there was a deterministic relation between higher CRP level, change in brain structure, and depression (D'Mello & Swain, 2017; Felger et al., 2020; Kelly et al., 2019; Ng et al., 2018; Vulser et al., 2015), still the relationship between peripheral inflammation, brain structure, and depression remains unclear. A recent work showed that DNA methylation of CRP was significantly associated with reduced global gray matter/cortical volume and widespread reductions in integrity of 16/24 white matter tracts, the methylation‐based measures showed stronger associations with imaging metrics than serum‐based CRP measures, and these findings provide evidence for central effects of peripheral inflammation from both serological and epigenetic markers of inflammation, including in brain regions previously implicated in depression (Green et al., 2020). A review demonstrated that the early life adversity–perinatal depressive risk model, as we have proposed, invokes epigenetic embedding as a key pathway that promotes a pro‐inflammatory phenotype (higher levels of pro‐inflammatory cytokines and lower levels of oxytocin), which, in turn, shapes maternal stress reactivity, mood, and behavior (Garfield et al., 2015). The present work preliminarily showed that higher CRP levels in hippocampus, prefrontal cortex, and hypothalamus were found in the PS susceptible offspring rats measured by immunohistochemistry, qRT‐PCR, and Western blotting, which provided an indirect explain for the depressive‐like behavior of offspring rats caused by PS. However, the direct role of CRP in the depressive‐like behavior is still need to be confirmed in future work.

In conclusion, PS induced depressive‐like behavior in susceptible offspring rats, and an increasing of CRP expression in hippocampus, prefrontal cortex, and hypothalamus. Thus, higher CRP expression in hippocampus, prefrontal cortex, and hypothalamus may play a critical role in the mechanism under depressive‐like behavior in offspring rats exposed to PS.

## CONFLICT OF INTEREST

The authors declare no competing interests.

## AUTHOR CONTRIBUTIONS

Shaoning Li and Hongli Sun contributed to the design of the study. Huifang Zhang and Xueyun Gao were involved with data acquisition. Xueyun Gao and Huimei Huang carried out the statistical analysis. Shaoning Li, Wei He, and Huiping Zhang drafted the manuscript. Hongli Sun revised the manuscript. All the authors assisted with interpretation of the results, contributed to revision of the manuscript, and approved the final version.

### PEER REVIEW

The peer review history for this article is available at https://publons.com/publon/10.1002/brb3.2046.

## Data Availability

The data that support the findings of this study are available from the corresponding author upon reasonable request.
